# The association of higher levels of within-normal-limits liver enzymes and the prevalence of the metabolic syndrome

**DOI:** 10.1186/1475-2840-9-30

**Published:** 2010-07-15

**Authors:** Arie Steinvil, Itzhak Shapira, Orit Kliuk Ben-Bassat, Michael Cohen, Yaffa Vered, Shlomo Berliner, Ori Rogowski

**Affiliations:** 1 Departments of Medicine "D" & "E", The Tel-Aviv Sourasky Medical Center, 6 Weizman St, Tel Aviv, 64239, Israel; 2 the Central Laboratory of the Tel Aviv Sourasky Medical Center, affiliated to the Sackler Faculty of Medicine, Tel Aviv University, Tel Aviv, Israel

## Abstract

**Background:**

Metabolic syndrome (MetS) is frequently characterized by elevated liver enzymes, including gamma-glutamyl transferase (GGT) and alanine aminotransferase (ALT). Our objective was to evaluate the range of prevalence of MetS in apparently healthy individuals whose liver enzyme concentrations were all within-normal-range.

**Methods:**

We have performed a cross sectional analysis on participants of the Tel-Aviv medical center inflammation survey (TAMCIS) recruited between the years 2003-2009. Analyzed were a cohort of 6,561 men and 3,389 women.

**Results:**

The prevalence of MetS increased significantly from the first quintile to the fifth for both GGT and ALT, all the five quintiles being in the normal range. Logistic regression analysis for the presence of MetS showed crude odds ratios of 2.7 and 2.4 between the first and fourth quintiles and 3.6 and 3.2 for the fifth quintile in men and women respectively for ALT. For GGT the respective odds being 3.6 and 3.2 for the fourth quintile and 3.9 and 3.4 for the fifth quintile in men and women, respectively.

**Conclusions:**

A relatively high prevalence of MetS was noted in a cohort of apparently healthy individuals with liver enzyme concentrations within-normal-limits. Practical consequences include the need to follow up these enzyme concentrations as continuous variables and to take into consideration that even relatively small elevations within the normal range might reflect the presence of dysmetabolism.

## Background

The metabolic syndrome (MetS) is a constellation of cardiovascular risk factors known to be associated with increased future atherothrombotic events [1-4]. Previous studies have shown that elevated liver enzymes concentrations were associated with the diagnosis of MetS and of its components [5-9].

Little is known however, about the association between the prevalence of the metabolic syndrome among individuals presenting with within-normal-range liver enzymes. To the best of our knowledge, there is only one paper which demonstrated that even 'normal' levels of ALT are associated with the long-term development of multiple metabolic disorders, including the MetS and diabetes mellitus [10].

Since both elevated GGT [11-18] and ALT [19-22] have demonstrated significant associations with both atherothrombotic burden and vascular risk, we have performed a cross sectional analysis to evaluate the prevalence of the MetS in a relatively large sample of apparently healthy individuals in whom both GGT and ALT as well as aspartate aminotransferase (AST) were all within the normal limits.

## Methods

### Study population

We have presently analyzed data that has been collected during the last five years in the Tel-Aviv medical center inflammation survey (TAMCIS), a registered data bank of the Israeli ministry of justice [23-29]. This is a relatively large cohort of individuals who attended our medical center for a routine annual check-up and who gave their written informed consent for participation according to the instructions of the institutional ethics committee. A total of 16,413 subjects gave their informed consent (10,389 males, 6,024 females). Later, 2,071 subjects were excluded from the analysis due to any malignancy or immunosuppressive therapy, pregnancy, steroidal or antibiotic treatment or recent acute infection. We further excluded 927 individuals due to diabetes mellitus and 221 individuals for reporting regular alcohol use of more than 1 glass per day. Finally, an additional 1,241 subjects were excluded for missing data on any of their liver function enzyme measurements and 2,003 individuals with any of the three liver function enzymes concentrations that we routinely check (ALT, GGT and AST) in whom these levels were found to be above the upper limit of normal according to our local laboratory. Following these exclusions the study group comprised 9,950 individuals (6,561 men and 3,389 women).

### Definitions of atherothrombotic risk factors

Results of the routine health check-up were assessed employing certain definitions in order to recognize atherothrombotic risk factors in individuals. These included diabetes mellitus which was defined as a fasting blood glucose concentration of ≥7.0 mmol/L or the intake of insulin or oral hypoglycemic medications. Hypertension was defined as a blood pressure of ≥ 140/90 mm Hg on two separate measurements or the use of antihypertensive medications. Dyslipidemia was defined as the low density lipoprotein cholesterol (LDL-C) or non-high density lipoprotein cholesterol (non-HDL-C) concentrations, for individuals displaying elevated triglyceride concentrations of > 2.26 mmol/L, above the recommended levels according to the risk profile defined by the updated adult treatment panel III (ATP III) recommendations [30] or the use of lipid lowering medications. The diagnosis of the metabolic syndrome was based on the joint interim statement of the International Diabetes Federation Task Force on Epidemiology and Prevention; National Heart, Lung, and Blood Institute; American Heart Association; World Heart Federation; International Atherosclerosis Society; and International Association for the Study of Obesity [31]. In short, elevated waist circumference was defined as ≥94 cm (37 inches) in men and ≥80 (31.5 inches) in women as recommended for europid and middle east; elevated triglycerides (TG) were defined as ≥150 mg/dl (1.7 mmol/l) or on drug treatment for elevated triglycerides; reduced HDL-C was defined as < 40 mg/dL (1.0 mmol/l) in men and < 50 mg/dl (1.3 mmol/l) in women or on drug treatment for reduced HDL-C; elevated blood pressure was defined as ≥130 mm Hg systolic blood pressure or ≥85 mm Hg diastolic blood pressure or on antihypertensive drug treatment in a patient with a history of hypertension; elevated fasting glucose was defined as ≥100 mg/dl (5.55 mmol/l). Smokers were defined as individuals who smoked at least 5 cigarettes per day while past smokers had quit smoking for at least 30 days prior to examination.

### Laboratory methods

All blood samples were drawn following a 12 hour fasting period. Glucose was measured by the glucose oxidase system [32]. We utilized an improved color reagent for the determination of blood glucose by the oxidase system using the Bayer Advia 1650 chemistry analyzer. Triglycerides were measured by an adaptation of the Fossati 3 step enzymatic reaction with the Bayer Advia 1650 chemistry analyzer. Serum triglycerides were determined calorimetrically with an enzyme that produces hydrogen peroxide[33]. HDL-C was determined by the method developed by Izawa et al [34] using the Bayer Advia 1650 chemistry analyzer. LDL-C was derived from the measured concentrations of total cholesterol, HDL-C and triglycerides using the Friedwald equation: LDL-C = Total Cholesterol - HDL-C - TG/5. GGT was determined by the method of Szasz [35] using the Bayer Advia 1650 chemistry analyzer. ALT was determined by the modified method in accordance with the International Federation of Clinical Chemistry (IFCC) [36] using the Bayer Advia 1650 chemistry analyzer.

### Statistical analysis

All data was summarized and displayed as mean (standard deviation [SD]) for the continuous variables and as number of patients (expressed as a percentage) in each group for the categorical variables. Since the triglyceride concentrations displayed irregular distributions, we used logarithmic transformation which converted the distributions to normal ones for all statistical procedures. Therefore all results of triglyceride concentrations are expressed as back-transformed geometrical means. The One-Way Kolmogorov-Smirnov test was used to assess the distributions. In order to characterize the population we divided the patients in each gender into quintiles of each of the two liver function enzymes of relevance, including GGT and ALT, and analyzed all results accordingly. For all categorical variables the Chi-square statistical test was used for assessing the statistical significance between the quintiles, while the One-Way Analysis of Variance was used for all continuous variables. In order to better appreciate the magnitude of the differences in the prevalence of the MetS between the quintiles of the liver enzymes and in order to adjust for differences in age, physical activity, smoking status and use of medications that are not a part of the parameters which define the MetS, we used logistic regression and calculated the odds ratio for having the MetS between the quintiles of each liver enzyme relative to the lower quintile. All above analyses were considered significant at p < 0.05 (two tailed). The SPSS statistical package was used to perform all statistical evaluations (SSPS Inc., Chicago, IL, USA).

## Results

**Figure 1:**
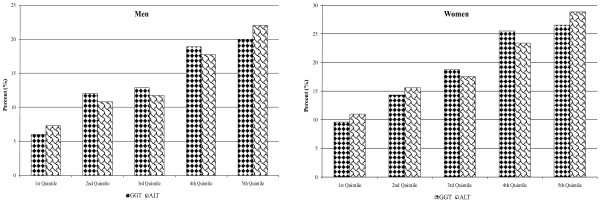
**Percentages of individuals with the metabolic syndrome (men and women) according to quintiles of each of the within-normal-limits liver enzyme concentrations (ALT, alanine aminotransferase; GGT, gamma-glutamyl transferase)**.

We have presently analyzed the results of a total of 6,561 men and 3,389 women at the mean (SD) age of 44 (11) years. Nine hundred and sixteen men (14%) and 645 women (19%) were characterized as having the metabolic syndrome. Epidemiological data as well as relevant biochemistry for men and women are given in Tables 1 and 2 for ALT and GGT, respectively. In Tables 3 and 4 we report the results of the logistic regression analyses for the presence of the MetS in relation to the quintiles of ALT and GGT, respectively. It can be seen that for ALT, the crude odds ratios increase up to 2.7 and 2.4 in the fourth quintile and even 3.6 and 3.2 for the fifth quintile for both men and women. The respective odds ratios for GGT being 3.6 and 3.2 in the fourth quintile and 3.9 and 3.4 for the fifth. The percentages of individuals with the MetS according to quintiles of each of the within-normal-limits liver enzyme concentrations are reported in Figure 1 for both men and women. Tables 3 &4 also evaluate the adjusted odds ratios after adjustment for several relevant parameters that are not a direct part of the definition of the MetS. Those parameters include age, exercise intensity, use of aspirin and statins, smoking status and alcohol consumption. We further adjusted our models to obesity and the results did not change significantly following all those adjustments. Finally, in order to decrease the influence of possible confounders, we have repeated this analysis by further excluding subjects receiving statins, fibrates, amiodarone as well as women taking oral contraceptives or hormonal replacement therapy and the results were not changed significantly (data not shown).

**Table 1:** Patient characteristics* according to quintiles of ALT

Men (N = 6561)	1^st ^Quintile		2^nd ^Quintile		3^rd ^Quintile		4^th ^Quintile		5^th ^Quintile		ANOVA/χ^2^	P for linear trend
	ALT<16		16≤ALT<20		20≤ALT<23		23≤ALT<29		29≤ALT≤40			
N	1196		1545		1121		1460		1239		P Value	
Age (years)	44	(11)	44	(11)	44	(11)	44	(11)	44	(11)	0.757	0.280
Waist (cm)	94	(10)	95	(11)	95	(11)	95	(10)	95	(10)	0.299	0.032
BMI (kg/m^2^)	27	(4)	27	(4)	27	(4)	27	(4)	27	(4)	0.084	0.017
Diastolic BP (mmHg)	78	(9)	78	(8)	78	(8)	78	(9)	79	(8)	0.206	0.025
Systolic BP (mmHg)	125	(15)	124	(14)	124	(15)	124	(15)	125	(14)	0.603	0.865
Alcohol consumption (glasses/week)	1.0	(1.5)	1.1	(1.6)	1.1	(1.5)	1.1	(1.6)	0.9	(1.5)	0.132	0.189
Exercise intensity (hours/week)	2.4	(3.0)	2.3	(2.7)	2.2	(2.5)	2.3	(2.9)	2.2	(2.5)	0.274	0.102
Smoking status, n (%)											0.685	
Current	184	(15.7)	256	(17.0)	177	(16.0)	215	(15.0)	207	(17.1)		
Former	292	(24.9)	396	(26.2)	286	(25.9)	388	(27.1)	328	(27.1)		
Hypertension, n (%)	286	(23.9)	362	(23.4)	298	(26.6)	340	(23.3)	328	(26.5)	0.111	
Anti-hypertensive medications, n(%)	100	(8.4)	145	(9.4)	118	(10.5)	133	(9.1)	135	(10.9)	0.192	
Metabolic Syndrome, n (%)	87	(7.3)	167	(10.8)	131	(11.7)	258	(17.7)	273	(22.0)	< 0.001	
Glucose (mmol/L)	4.86	(0.45)	4.99	(0.50)	5.07	(0.52)	5.13	(0.53)	5.19	(0.55)	< 0.001	< 0.001
HDL Cholesterol (mmol/L)	1.57	(0.39)	1.50	(0.37)	1.46	(0.36)	1.39	(0.33)	1.34	(0.31)	< 0.001	< 0.001
LDL Cholesterol (mmol/L)	2.90	(0.81)	3.07	(0.82)	3.11	(0.80)	3.20	(0.82)	3.21	(0.80)	< 0.001	< 0.001
Triglycerides^† ^(mmol/L)	0.95		1.00		1.09		1.21		1.35		< 0.001	< 0.001
Statins, n(%)	117	(9.8)	142	(9.2)	97	(8.7)	129	(8.8)	124	(10.0)	0.732	
Fibrates, n(%)	14	(1.2)	19	(1.2)	11	(1.0)	12	(0.8)	10	(0.8)	0.718	

**Women (N = 3389)**	**1^st ^Quintile**		**2^nd ^Quintile**		**3^rd ^Quintile**		**4^th ^Quintile**		**5^th ^Quintile**		**ANOVA**	**P for linear trend**
	**ALT<16**		**16≤ALT<19**		**19≤ALT<22**		**22≤ALT<27**		**27≤ALT≤35**		**P Value**	
**N =**	**767**		**652**		**607**		**738**		**625**			

Age (years)	44	(11)	45	(11)	45	(11)	45	(11)	45	(11)	0.703	0.369
Waist (cm)	82	(12)	82	(12)	82	(12)	82	(12)	83	(11)	0.389	0.171
BMI (kg/m^2^)	25	(5)	25	(5)	25	(5)	25	(5)	26	(5)	0.489	0.248
Diastolic BP (mmHg)	73	(8)	74	(8)	74	(8)	74	(9)	74	(8)	0.209	0.019
Systolic BP (mmHg)	117	(15)	117	(16)	117	(15)	117	(16)	118	(16)	0.688	0.420
Alcohol consumption (glasses/week)	0.5	(1.1)	0.5	(1.1)	0.5	(1.1)	0.5	(1.2)	0.5	(1.1)	0.999	0.867
Exercise intensity (hours/week)	1.9	(2.8)	1.8	(2.5)	2.0	(3.4)	1.7	(2.3)	1.8	(2.4)	0.474	0.329
Smoking status, n (%)											0.447	
Current	150	(19.9)	129	(20.3)	99	(16.6)	136	(18.8)	114	(18.7)		
Former	145	(19.2)	124	(19.5)	131	(22.0)	125	(17.3)	126	(20.7)		
Hypertension, n (%)	106	(13.8)	111	(17.0)	89	(14.7)	118	(16.0)	101	(16.2)	0.481	
Anti-hypertensive medications, n(%)	55	(7.2)	56	(8.6)	46	(7.6)	52	(7.0)	40	(6.4)	0.648	
Metabolic Syndrome, n (%)	84	(11.0)	102	(15.6)	106	(17.5)	173	(23.4)	180	(28.8)	< 0.001	
Glucose (mmol/L)	4.87	(0.48)	4.95	(0.50)	5.05	(0.53)	5.12	(0.55)	5.18	(0.55)	< 0.001	< 0.001
HDL Cholesterol (mmol/L)	1.60	(0.37)	1.52	(0.40)	1.48	(0.37)	1.41	(0.35)	1.35	(0.33)	< 0.001	< 0.001
LDL Cholesterol (mmol/L)	2.90	(0.80)	3.02	(0.80)	3.13	(0.79)	3.19	(0.81)	3.15	(0.85)	< 0.001	< 0.001
Triglycerides^† ^(mmol/L)	0.93		1.01		1.06		1.21		1.29		< 0.001	< 0.001
Statins, n(%)	59	(7.7)	40	(6.1)	48	(7.9)	41	(5.6)	33	(5.3)	0.164	
Fibrates, n(%)	6	(0.8)	5	(0.8)	5	(0.8)	0	(0)	1	(0.2)	0.073	

* - Data are arithmetic means (S.D.) or N (%).

† - Geometric means.

List of Abbreviations: ALT, alanine aminotransferase; BMI, body mass index; BP, blood pressure; HDL, high density lipoprotein; LDL, low density lipoprotein.

**Table 2:** Patients characteristics* according to quintiles of GGT

Men (N = 6561)	1^st ^Quintile		2^nd ^Quintile		3^rd ^Quintile		4^th ^Quintile		5^th ^Quintile		ANOVA	P for linear trend
	GGT<9		9≤GGT<12		12≤GGT<16		16≤GGT<21		21≤GGT≤42		P Value	
N =	1318		1206		1443		1202		1392			
Age (years)	44	(11)	44	(11)	44	(11)	44	(11)	44	(11)	0.482	0.848
Waist (cm)	95	(10)	95	(11)	95	(11)	95	(10)	95	(11)	0.721	0.165
BMI (kg/m^2^)	27	(4)	27	(4)	27	(4)	27	(4)	27	(4)	0.875	0.433
Diastolic BP (mmHg)	78	(8)	78	(8)	78	(9)	78	(9)	78	(9)	0.398	0.063
Systolic BP (mmHg)	124	(14)	125	(15)	123	(14)	125	(15)	124	(14)	0.062	0.244
Alcohol consumption (glasses/week)	1.1	(1.6)	1.0	(1.5)	1.0	(1.5)	1.1	(1.6)	1.0	(1.6)	0.458	0.466
Exercise intensity (hours/week)	2.4	(2.9)	2.2	(2.6)	2.3	(2.8)	2.2	(2.7)	2.3	(2.5)	0.327	0.254
Smoking status, n (%)											0.785	
Current	205	(15.9)	194	(16.3)	217	(15.4)	198	(16.8)	225	(16.5)		
Former	333	(25.9)	313	(26.4)	368	(26.1)	332	(28.1)	344	(25.2)		
Hypertension, n (%)	301	(22.8)	298	(24.7)	347	(24.0)	329	(27.4)	339	(24.4)	0.113	
Anti-hypertensive medications, n(%)	120	(9.1)	117	(9.7)	131	(9.1)	122	(10.1)	141	(10.1)	0.790	
Metabolic Syndrome, n (%)	79	(6.0)	145	(12.0)	186	(12.9)	227	(18.9)	279	(20.0)	< 0.001	
Glucose (mmol/L)	4.87	(0.47)	4.95	(0.49)	5.06	(0.52)	5.15	(0.54)	5.20	(0.53)	< 0.001	< 0.001
HDL Cholesterol (mmol/L)	1.58	(0.39)	1.48	(0.38)	1.44	(0.34)	1.39	(0.34)	1.36	(0.32)	< 0.001	< 0.001
LDL Cholesterol (mmol/L)	2.93	(0.79)	3.05	(0.83)	3.11	(0.82)	3.21	(0.82)	3.21	(0.79)	< 0.001	< 0.001
Triglycerides^† ^(mmol/L)	0.92		1.02		1.08		1.23		1.35		< 0.001	< 0.001
Statins, n(%)	111	(8.4)	95	(7.9)	114	(7.9)	129	(10.7)	160	(11.5)	0.001	
Fibrates, n(%)	9	(0.7)	13	(1.1)	12	(0.8)	20	(1.7)	12	(0.9)	0.116	

**Women (N = 3389)**	**1^st ^Quintile**		**2^nd ^Quintile**		**3^rd ^Quintile**		**4^th ^Quintile**		**5^th ^Quintile**		**ANOVA**	**P for linear trend**
	**GGT<8**		**8≤GGT<11**		**11≤GGT<15**		**15≤GGT<19**		**19≤GGT≤28**		**P Value**	
**N =**	**586**		**670**		**846**		**620**		**667**			

Age (years)	45	(10)	46	(11)	44	(11)	45	(10)	44	(11)	0.001	0.013
Waist (cm)	81	(11)	83	(12)	82	(12)	83	(11)	82	(12)	0.037	0.697
BMI (kg/m^2^)	25	(5)	26	(5)	25	(5)	26	(5)	25	(5)	0.047	0.636
Diastolic BP (mmHg)	73	(7)	74	(8)	74	(8)	74	(8)	73	(9)	0.028	0.323
Systolic BP (mmHg)	117	(15)	118	(16)	117	(16)	117	(15)	116	(15)	0.066	0.111
Alcohol consumption (glasses/week)	0.4	(1.0)	0.4	(1.0)	0.5	(1.2)	0.5	(1.1)	0.6	(1.2)	0.098	0.058
Exercise intensity (hours/week)	1.8	(2.7)	1.9	(2.6)	1.9	(2.7)	1.8	(3.1)	1.8	(2.3)	0.869	0.802
Smoking status, n (%)											0.515	
Current	107	(18.8)	137	(20.9)	165	(19.9)	106	(17.5)	113	(17.2)		
Former	105	(18.5)	127	(19.3)	150	(18.1)	130	(21.5)	139	(21.1)		
Hypertension, n (%)	91	(15.5)	117	(17.5)	127	(15.0)	103	(16.6)	87	(13.0)	0.216	
Anti-hypertensive medications, n(%)	53	(9.0)	51	(7.6)	59	(7.0)	51	(8.2)	35	(5.2)	0.101	
Metabolic Syndrome, n (%)	56	(9.6)	96	(14.3)	158	(18.7)	158	(25.5)	177	(26.5)	< 0.001	
Glucose (mmol/L)	4.83	(0.48)	4.96	(0.51)	5.03	(0.53)	5.15	(0.56)	5.16	(0.54)	< 0.001	< 0.001
HDL Cholesterol (mmol/L)	1.63	(0.38)	1.52	(0.38)	1.46	(0.37)	1.40	(0.34)	1.40	(0.36)	< 0.001	< 0.001
LDL Cholesterol (mmol/L)	2.87	(0.77)	3.03	(0.82)	3.11	(0.78)	3.20	(0.85)	3.15	(0.83)	< 0.001	< 0.001
Triglycerides^† ^(mmol/L)	0.90		0.96		1.10		1.21		1.31		< 0.001	< 0.001
Statins, n(%)	40	(6.8)	58	(8.7)	43	(5.1)	44	(7.1)	36	(5.4)	0.046	
Fibrates, n(%)	4	(0.7)	6	(0.9)	2	(0.2)	1	(0.2)	4	(0.6)	0.265	

* - Data are arithmetic means (S.D.) or N (%).

† - Geometric means.

List of abbreviations: GGT, gamma-glutamyl transferase; BMI, body mass index; BP, blood pressure; HDL, high density lipoprotein; LDL, low density lipoprotein.

**Table 3:** Logistic regression analysis for the presence of MetS in relation ALT quintiles*.

Men (N = 6561)	1^st ^Quintile	2^nd ^Quintile	3^rd ^Quintile	4^th ^Quintile	5^th ^Quintile
	ALT<16	16≤ALT<20	20≤ALT<23	23≤ALT<29	29≤ALT≤40
N	1196	1545	1121	1460	1239
Crude OR	1.0	1.5 (1.2 - 2.0)	1.7 (1.2 - 2.2)	2.7 (2.1 - 3.5)	3.6 (2.8 - 4.6)
Age adjusted OR	1.0	1.5 (1.2 - 2.0)	1.7 (1.3 - 2.3)	2.7 (2.1 - 3.5)	3.7 (2.8 - 4.8)
Multi-adjusted^† ^OR	1.0	1.6 (1.2 - 2.1)	1.7 (1.3 - 2.3)	2.8 (2.1 - 3.6)	3.7 (2.8 - 4.8)
Multi-adjusted + waist & BMI OR	1.0	1.5 (1.2 - 2.0)	1.7 (1.2 - 2.2)	2.8 (2.2 - 3.7)	3.7 (2.8 - 4.9)

**Women (N = 3389)**	**1^st ^Quintile**	**2^nd ^Quintile**	**3^rd ^Quintile**	**4^th ^Quintile**	**5^th ^Quintile**
	**ALT<16**	**16≤ALT<19**	**19≤ALT<22**	**22≤ALT<27**	**27≤ALT≤35**
**N =**	**767**	**652**	**607**	**738**	**625**

Crude OR	1.0	1.5 (1.1 - 2.0)	1.7 (1.2 - 2.3)	2.4 (1.8 - 3.2)	3.2 (2.4 - 4.2)
Age adjusted OR	1.0	1.4 (1.1 - 2.0)	1.6 (1.2 - 2.3)	2.5 (1.9 - 3.4)	3.3 (2.4 - 4.4)
Multi-adjusted^† ^OR	1.0	1.5 (1.1 - 2.0)	1.6 (1.2 - 2.2)	2.5 (1.9 - 3.4)	3.3 (2.5 - 4.4)
Multi-adjusted + waist & BMI OR	1.0	1.4 (1.0 - 2.0)	1.7 (1.2 - 2.3)	2.6 (1.9 - 3.5)	3.3 (2.4 - 4.5)

* The lower quintile was arbitrarily defined as 1.0

† Multi-adjusted means adjustments were made for age, exercise intensity, use of aspirin and statins, smoking status and alcohol consumption.

List of Abbreviations: ALT, alanine aminotransferase; MetS, metabolic syndrome; OR, odds ratio.

**Table 4:** Logistic regression analysis for the presence of MetS in relation GGT quintiles*.

Men (N = 6561)	1^st ^Quintile	2^nd ^Quintile	3^rd ^Quintile	4^th ^Quintile	5^th ^Quintile
	GGT<9	9≤GGT<12	12≤GGT<16	16≤GGT<21	21≤GGT≤42
N =	1318	1206	1443	1202	1392
Crude OR	1.0	2.1 (1.6 - 2.8)	2.3 (1.8 - 3.0)	3.6 (2.7 - 4.7)	3.9 (3.0 - 5.1)
Age adjusted OR	1.0	2.2 (1.6 - 2.9)	2.4 (1.8 - 3.2)	3.7 (2.8 - 4.8)	4.1 (3.1 - 5.3)
Multi-adjusted^† ^OR	1.0	2.2 (1.6 - 2.9)	2.4 (1.8 - 3.2)	3.7 (2.8 - 4.8)	4.1 (3.1 - 5.3)
Multi-adjusted + waist & BMI OR	1.0	2.3 (1.7 - 3.0)	2.5 (1.9 - 3.3)	4.0 (3.0 - 5.3)	4.5 (3.4 - 5.9)

**Women (N = 3389)**	**1^st ^Quintile**	**2^nd ^Quintile**	**3^rd ^Quintile**	**4^th ^Quintile**	**5^th ^Quintile**
	**GGT<8**	**8≤GGT<11**	**11≤GGT<15**	**15≤GGT<19**	**19≤GGT≤28**
**N =**	**586**	**670**	**846**	**620**	**667**

Crude OR	1.0	1.6 (1.1 - 2.3)	2.2 (1.6 - 3.0)	3.2 (2.3 - 4.4)	3.4 (2.5 - 4.7)
Age adjusted OR	1.0	1.5 (1.1 - 2.2)	2.3 (1.7 - 3.3)	3.3 (2.4 - 4.7)	3.9 (2.8 - 5.4)
Multi-adjusted^† ^OR	1.0	1.5 (1.1 - 2.2)	2.4 (1.7 - 3.3)	3.3 (2.4 - 4.7)	3.9 (2.8 - 5.5)
Multi-adjusted + waist & BMI OR	1.0	1.4 (1.0 - 2.1)	2.4 (1.7 - 3.4)	3.4 (2.4 - 4.7)	4.1 (2.9 - 5.8)

* The lower quintile was arbitrarily defined as 1.0.

† Multi-adjusted means adjustments were made for age, exercise intensity, use of aspirin and statins, smoking status and alcohol consumption.

List of abbreviations: GGT, gamma-glutamyl transferase; MetS, metabolic syndrome; OR, odds ratio.

## Discussion

We have presently shown that the MetS is prevalent in individuals who present normal concentrations of liver enzymes. Special attention has been given to two of them (GGT and ALT) for whom epidemiological evidence suggests an association with increased vascular risk. Goessling et al [10] have recently studied the relationship between aminotransferase levels and the incidence of MetS, DM, cardiovascular disease and all cause mortality in long-term follow-up of 2812 participants in the community-based Framingham Heart Study sample. They demonstrated a clear association between elevated ALT levels and the development of MetS and DM over 20 years of follow-up. They also found that ALT levels within the normal range are associated with adverse metabolic outcomes [10].

In our study, we found that the prevalence of the MetS doubles if a comparison is made between the first, second and third quintiles of both GGT and ALT. In these quintiles the concentrations of the enzymes are not only regarded as being absolutely normal, but are actually even in the lower range of the "normal" values. Assuming a link between these enzymes and a potential dysmetabolic state, the finding might suggest a relatively high sensitivity of these biomarkers to reveal the presence of dysmetabolism.

Elevated liver enzymes are well documented in individuals with the MetS and are markers of fatty liver changes [37]. To a certain degree these changes can be visualized by the use of ultrasonography but this procedure is not cost effective and lacks sufficient sensitivity and specificity on a population level in screening apparently healthy individuals with within-normal-limits concentrations of liver enzymes. It would certainly not normally be performed in asymptomatic individuals whose liver enzymes are in the lower part of the so-called normal range and, if performed, would barely reveal the typical changes if at all. Therefore, quantitative analysis of liver enzymes remains the main diagnostic tool in this situation.

The present findings are not necessarily of solely theoretical relevance. In fact, the elevations in the concentrations of these liver enzymes might be contributory in the etiopathogenesis of the atherothrombotic inflammatory process. In particular, GGT might have an important role in the extracellular catabolism of glutathione, the principal thiol antioxidant in humans [14]. In addition, this enzyme has the ability to catalyze the oxidation of LDL-C [38] and may also be pro-inflammatory due to the fact that it mediates the interconversion of leukotriene C4 into D4 [39]. As mentioned, GGT may directly take part in atherogenesis and evolve as a potential biochemical risk indicator of cardiovascular morbidity and mortality and thus have an application in primary and secondary prevention of cardiovascular disease [18]. Alanine aminotransferase however, is considered a more liver-specific marker than GGT. This is due to the fact that GGT is present on the surface of most cell types and is highly active in organs other than the liver [18]. Although both ALT and GGT have shown predictive for diabetes in a recent meta-analysis, GGT may be a better diabetes predictor than ALT [19]. In this regard, an increased enzyme level in the absence of known liver disease most commonly reflect liver fat deposition and is representative of the presence of visceral fat [21]. This visceral fat and obesity is central to the nuclear peroxisome proliferator activated receptors (PPAR) deactivation contributing to the development of insulin resistance, MetS and atherothrombosis [40,41].

The notion that persistently elevated liver enzyme concentrations, even within the so-called "reference" range, is associated with cardiovascular risk factors and future disease has been previously demonstrated [42]. In fact, it has been shown that several liver enzymes, in particular GGT, are associated with the appearance of cardiovascular events [13]. This is also relevant to individuals with the MetS [14]. Thus, the recognition that the prevalence of the MetS increases even in individuals whose liver enzymes are in the lower part of the 'normal' reference range, might have clinical significance in terms of early recognition of these dysmetabolic changes.

We recognize several limitations in the current analysis. First, our conclusions are of a descriptive nature and are based essentially only on associations between parameters. Based upon its cross sectional design, the present findings are inherently limited in the ability to eliminate causal relationships. Second, since some of the study population had several risk factors, we could not fully eliminate the possible effect of underlying diseases and medications used for these diseases on the present findings. Further prospective population-based studies are needed to investigate the mechanisms in order to answer these questions. Finally, serum GGT is a sensitive indicator of alcohol consumption and/or liver dysfunction such as fatty liver, and is also high in patients with liver disease: chronic viral hepatitis, primary biliary cirrhosis, or drug-induced liver injury. These liver diseases are present in community-dwelling persons and are usually asymptomatic. Thus, these possible confounders could have affected results.

We conclude that a relatively high prevalence of the MetS has been noted in a cohort of apparently healthy individuals who have within-normal-limits concentrations of liver enzymes. The relatively high prevalence of the MetS observed could explain, at least in part, the previously noted epidemiological associations between elevated concentrations of GGT and ALT and future vascular risk. Moreover, our findings suggest that even minute changes, still within the so called "normal range" could point towards a potential dysmetabolic state. These observations could therefore lead to avoiding the usage of cut-off values for normalcy for these two biomarkers. In addition, they should encourage the use of both GGT and ALT as continuous biomarkers that could be used for early signaling of dysmetabolism. Finally, our results support the notion that in the era of early detection, prevention and treatment of metabolic disorders, one cannot be confident that relatively low concentrations of liver enzymes exclude the presence of dysmetabolic changes. A practical consequence might therefore be to follow these enzyme concentrations as continuous biomarkers and take into consideration the possibility that even small changes in their concentrations might be of relevance.

## Declaration of competing interests

The authors declare that they have no competing interests.

## Authors' contributions

OR and AS have participated in the design of the study, performed the statistical analyses and drafted the paper. SB and IS conceived the study, participated in its design and coordination and helped to draft and review the manuscript. OKB, MC and YV helped in the data organization and retrieval, English editing and final draft preparation. All of the authors have read and approved the final manuscript.
